# Liquid Temperature Measurements Using Two Different Tunable Hollow Prisms

**DOI:** 10.3390/s17020266

**Published:** 2017-01-29

**Authors:** Sergio Calixto, Martha Rosete-Aguilar, Ismael Torres-Gomez

**Affiliations:** 1 Centro de Investigaciones en Optica, Loma del Bosque 115, Leon 37150, Mexico; itorres@cio.mx; 2 Centro de Ciencias Aplicadas y Desarrollo Tecnológico (CCADET), Universidad Nacional Autónoma de México (UNAM), Av. Universidad 3000, Coyoacán, Distrito Federal 04510, Mexico; martha.rosete@ccadet.unam.mx

**Keywords:** temperature sensors, grism, constant deviation prism, Pellin-Broca

## Abstract

This paper describes the design, fabrication, and testing of two hollow prisms. One is a prism with a grating glued to its hypotenuse. This ensemble, prism + grating, is called a grism. It can be applied as an on-axis tunable spectrometer. The other hollow prism is a constant deviation one called a Pellin-Broca. It can be used as a tunable dispersive element in a spectrometer with no moving parts. The application of prisms as temperature sensors is shown.

## 1. Introduction

A grism [1] is an optical structure that has a prism and a grating arranged to keep light, with a given wavelength, undeviated as it passes through the grism. The grating is placed in the hypotenuse of the prism. This grating can be ruled directly into the hypotenuse or it can be replicated with the help of a resin and glued to the prism. Grisms are used in direct vision spectroscopes and are usually inserted into a collimated beam. Grisms are used in astronomy to create a dispersed spectrum centered on the location of the object in the camera field of view. With this method cameras are converted to spectrographs. Grisms are also used as pulse shapers and for pulse compression [2] and dispersion compensation of femtosecond pulses.

In astronomy it is common to use several grisms to cover different parts of the spectrum. For example the Near Infrared Camera and Multiobject Spectrometer (NICMOS) [3], in the Hubble Space Telescope, uses three grisms (G096, G141, and G206) to cover a spectral range between 0.8 µm to 2.4 µm. Another use of grisms was made in the First Light Test Experiment Camera (FLITECAM) [4] that is in the NASA’s SOFIA aircraft. There are three grisms made of KRS-5 and each has a direct-ruling grating. With these grisms it is possible to make observations in nine spectral bands covering a spectral range from about 1 µm to 5.5 µm. Regarding the ultraviolet region of the spectrum, grisms have been fabricated for NASA’s ultraviolet optical telescope (UVOT) [5]. Two grisms cover the spectral bands from about 200 nm to 300 nm and from 300 nm to about 400 nm. An example of a grism working with visible light is the one used at the Nordic Optical Telescope (NOT) [6]. The grism works in the band 345–515 nm, at first order.

As we can see from the description in the above paragraph several grisms are needed if a wide spectral band is to be covered. Here we suggest the use of only one hollow grism that could cover a spectral band in the visible region. This grism is hollow and different liquids are poured in it to tune the spectral region. The very first results of this research with the grism were presented in a conference paper [7].

In addition to the grism, we present a hollow constant deviation tunable prism (Pellin-Broca). When an equilateral prism (60°) is used in a spectrometer the incoming collimated light passes through the prism and then through a telescope that collects the light. The prism should be rotated to use it at minimum deviation. Then the telescope rotates to find the desired wavelength. On the other hand, in a constant deviation spectroscope [8], the collimator and the telescope are fixed at right angles and the prism is rotated to select a wavelength. Here we suggest and show that a hollow constant deviation prism, Pellin-Broca, can be used in the spectrometer. With this hollow prism it is not necessary to rotate it to select the spectral line, just that the liquid in the hollow prism should be changed.

Nowadays there are electronic thermometers [9] like those based on platinum, cooper-nickel, rhodium-iron, and germanium, to mention but a few. Additionally, there are those based on semiconducting ceramic, like the thermistor, with a sensitivity of 50 mV/°C. This sensitivity is 100 times that of platinum and 1000 times that of thermocouples. However, thermistors show disadvantages, like extreme non-linearity of resistance with temperature and instability with time and cycling. Additionally, electronic thermometers are affected by electromagnetic fields, and at the same time, influence them.

Optical thermometers [10] are immune to electric and magnetic fields; thus, they can be employed in the temperature measurement in induction, dielectric, and microwave heating because they are made of isolating materials and may contact live metallic elements. They present immunity to chemical and mechanical influences. Some optical detectors, based on fiber optics [11], can present high resolution (6 × 10^−4^ °C), fast response time (0.5 ms), and sensitivity of 84 pm/°C.

After showing the characterization of a grism and a Pellin-Broca we present their use as optical thermometers. This is based on the liquid density change, due to temperature, that will give a change to the refractive index.

In Section 2 we describe the principle of the grism method. Section 3 exposes the grism fabrication method. Section 4 shows the simulation or modeling of the grism with an optical design program. Section 5 describes the testing of the grism. Section 6 shows the physical principle of the Pellin-Broca prism and the fabrication method. Section 7 describes the testing of the Pellin-Broca. Section 8 shows the application of hollow prisms to the measurement of temperature.

It should be mentioned that the elements like the prisms, grating pitch, fibers, light sources, and other optical elements in the optical configurations were used because of their availability. However, the optical configurations are versatile and the selection of optical components with different characteristics can be used in order to improve their application.

## 2. Physical Principle of the Method

**Figure 1:**
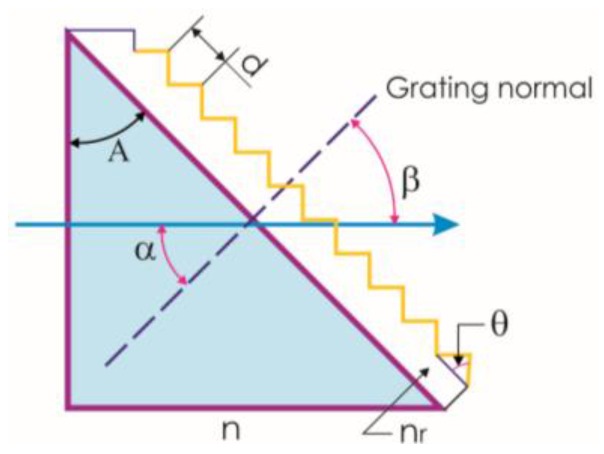
Description of a solid grism.

A solid grism consists of a prism and a grating. Figure 1 shows the structure [1]. Parameters that should be considered are the prism angle (*A*), the grating pitch (*d*), the prism refractive index (*n*), the resin refractive index (*n*_r_), the grating facet angle (θ), the angle that makes the light with the grating normal (α), and the angle that makes the diffracted beam (β). The grating equation applied to a grism is the following: *mλ* = (*n*sinα + *n*′sinβ). Supposing the grism is immersed in air, then *n*’ = 1 and α = −β = *A*, and the equation becomes:

*mλ* = *d*·(*n* − 1)·sin*A* (1)

Thus, light with a wavelength *λ* will pass through the prism undeviated. If we require light with a different wavelength with which the grism was designed to pass undeviated we could change the prism angle *A*, or the grating pitch (*d*), or the prism refractive index (*n*). We have chosen to make a hollow prism and fill it with liquids having different refractive indices; that is, *n* is variable.

## 3. Tunable Grism Fabrication Method

To make a tunable grism we need two optical structures: a hollow prism and a blazed grating. Due to the grating characteristics the angle A was calculated to be 46°. More on these details will be exposed below.

**Figure 2:**
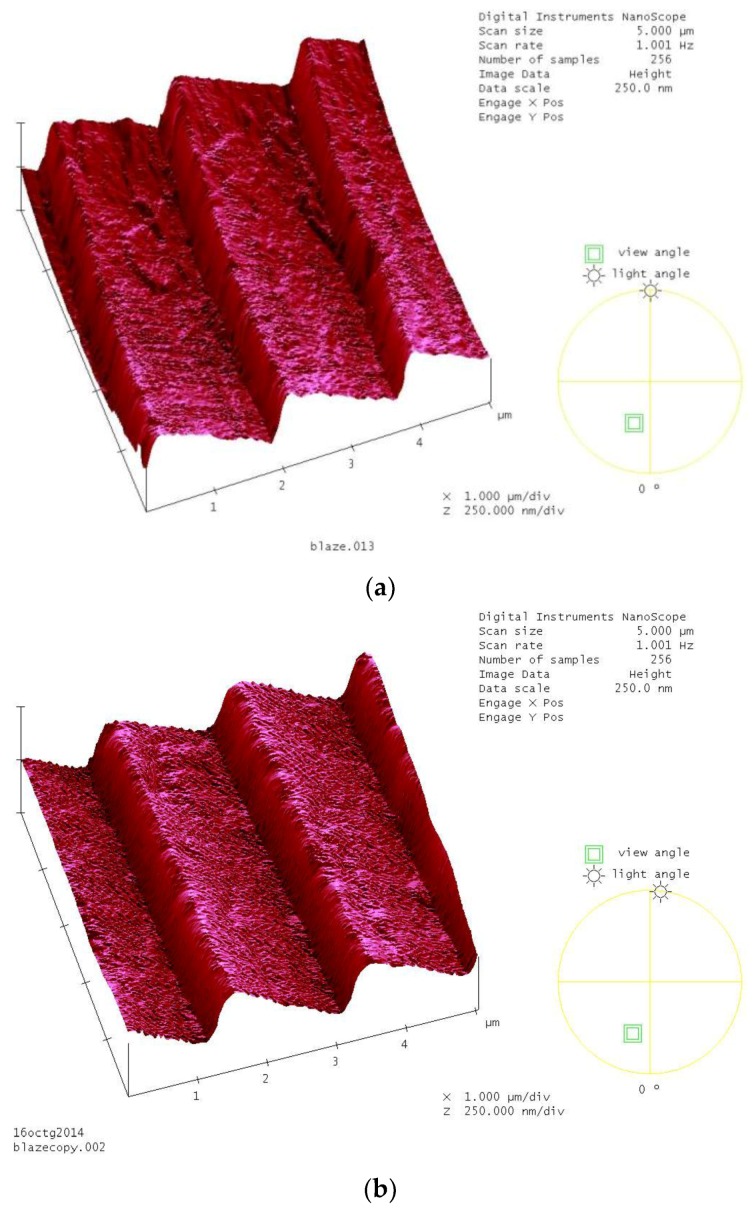
Surfaces of the master grating (**a**) and a copy of it in a silicone material (**b**). Images given by AFM.

**Figure 3:**
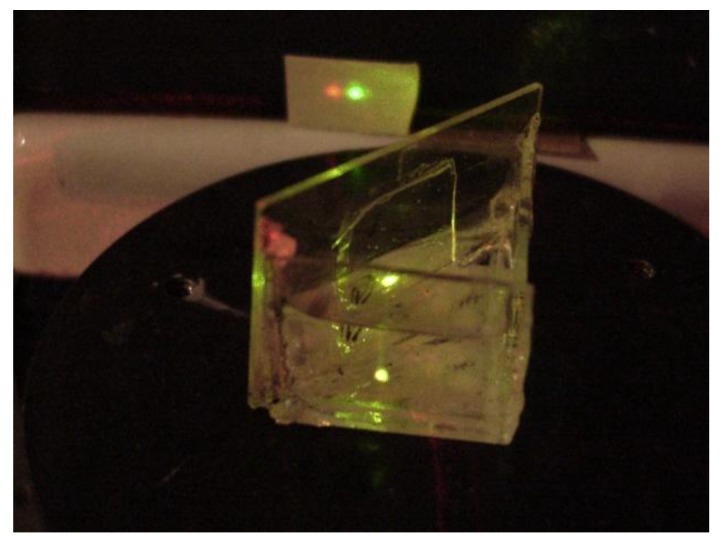
A photograph showing one of the fabricated grisms. On the first plane it is possible to see a red and a green beam, superposed, entering the grism face. Then follows the liquid in the cell. At the hypotenuse of the grism the glued grating can be seen. In the background two spots of light illuminate a white screen: the red spot on the left side and the green on the right side. They are shifted to the left because the grism was designed to let light with a wavelength of 400 nm pass through directly.

**Figure 4:**
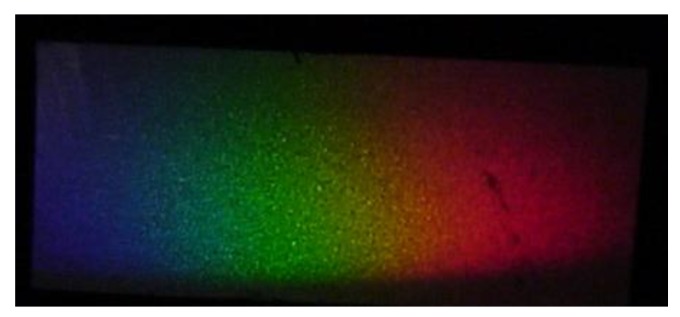
The spectrum given by the grism on ground glass. Its longer size is about 1 cm.

The grating fabrication method is as follows: A blazed master grating having a pitch of *d* = 1.69 µm and a groove angle of 8° was chosen. Liquid silicone was poured over its surface and, after polymerization, the thin film was detached from the master grating surface. This silicone grating was glued to the grism hypotenuse. Figure 2a shows the master grating surface and Figure 2b shows the silicone grating surface when they were studied with an atomic force microscope (AFM). Figure 3 shows the fabricated tunable hollow grism. Figure 4 shows the spectrum given by the grism.

## 4. Grism Simulation

**Figure 5:**
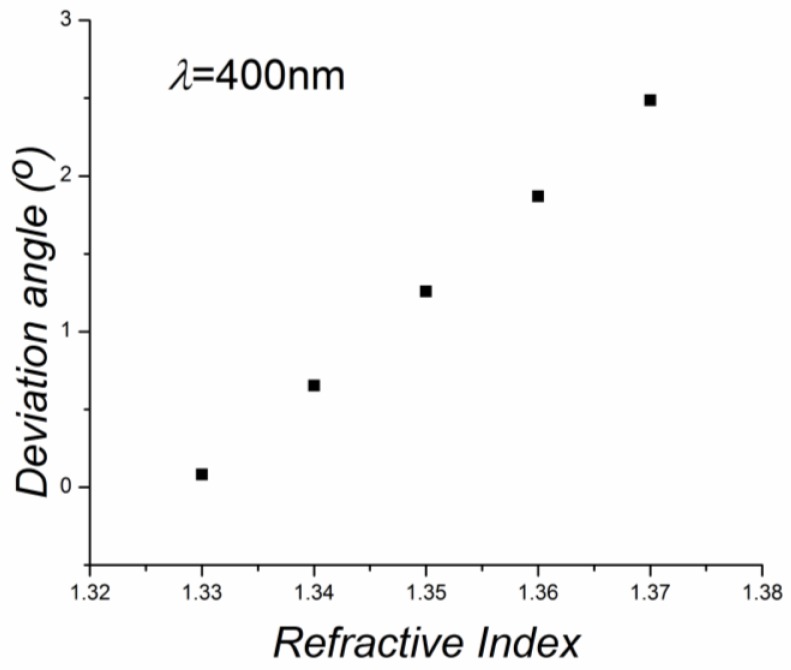
Calculated deviation angle as a function of the refractive index when a grism was used.

The behavior of the tunable hollow grism structure was simulated with an optical design program. Parameters that were considered are: the refractive index of the glass plates, 1.5; glass plate thickness, 1 mm; light that illuminated the grism has a wavelength of 400 nm;, grating pitch, 1.69 µm; and the liquid inside the grism had a refractive index of 1.33 (water) at the beginning and, later, the following refractive indices were considered: 1.34, 1.35, 1.36, and 1.37. Results can be seen in Table 1. The first column shows the liquid refractive index, the second column shows the deviation angle for the first diffracted order. For the first liquid with a refractive index of 1.33 it is seen that light was slightly deviated from the optical axis. However, when the refractive index changed, the deviation angle increased. These data were plotted and shown in Figure 5.

## 5. Experimentally Verifying the Grism Tuning

**Figure 6:**
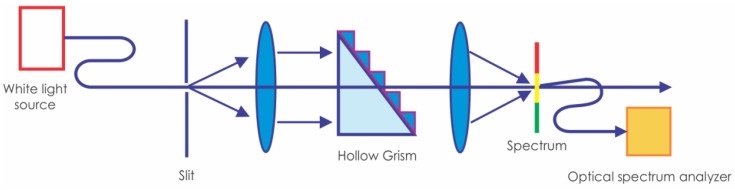
Optical configuration used to characterize the spectrum deviation when the liquid in the hollow Grism was changed.

To verify the beam deviation when the liquids with different refractive index are poured in the hollow grism, the optical configuration shown in Figure 6 was used. A white light source illuminates the setup. With the help of a fiber optic bundle a slit was illuminated. Then a lens collimated the white light beam. The beam traversed the grism and then a lens focused it. At the focal distance a white light spectrum was seen. An optical fiber was fixed and sampled part of the spectrum. Then, the signal was sent to an optical spectrum analyzer (OSA, AQ-6315 AB, ANDO Electric CO., Kanagawa Kawasaki, Japan,) to capture part of the spectrum. The fiber had a 105 µm core. A fiber with this size let us capture enough light to be sensed by the detector.

**Figure 7:**
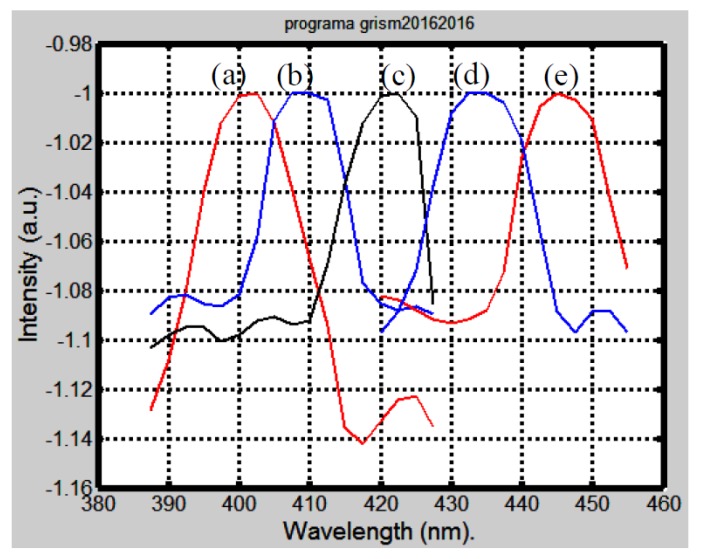
Plots showing the spectrum collected by an optical fiber. The parameter in the experiment was the refractive index in the hollow grism. The following refractive index values of the liquids injected into the hollow grism were used: (**a**) 1.33 (far left plot); (**b**) 1.34; (**c**) 1.35; (**d**) 1.36; (**e**) 1.37.

The spectrum was recorded every time a liquid with a different refractive index was poured in the hollow grism. Figure 7 shows the spectra. We notice that when a liquid with a refractive index of 1.33 was used, the spectrum showed a peak at 402 nm. Table 2 shows the relation between the refractive index that filled the hollow grism and the wavelength peak of the spectra (second column). Additional, Table 2 shows the wavelength for the beam that passes undeviated (third column). It was calculated with Equation (1). The last column shows the peak wavelength calculated with an optical design program. The agreement in columns three and four shows that the experimental grism can be described with Equation (1). In other words, the possible error introduced by the thickness of the glass plate is negligible. We can say that theoretical values agree with the experimental ones. Thus, we can say that it is possible to tune the wavelength that passes undeviated through the grism.

A note about the use of the grism as a spectrometer when room temperature changes during the measurement is shown below. Let us suppose that the liquid in the grism is water. We can calculate with the design program the wavelength that will be undeviated when it passes through the grism if the temperature changes from 10 °C to 80 °C. We consider the values of the water refractive indices as a function of temperature given in [12]. Table 3 gives the results. We can see that when room temperature changes from 10 °C to 80 °C there is a change of the peak wavelength of 13 nm (406.1 − 393.1 nm). Thus, the plots in Figure 7 will shift to shorter wavelengths due to the change in water temperature. However, when the temperature changes from 30 °C to 20 °C there is only a shift of 1.3 nm in the wavelength. Thus, if the grism is used as a spectrometer, and during the measurements the room temperature is kept between ±1 °C, the measurements will be affected by less than 1 nm.

## 6. Principle and Fabrication of the Pellin-Broca Tunable Prism

**Figure 8:**
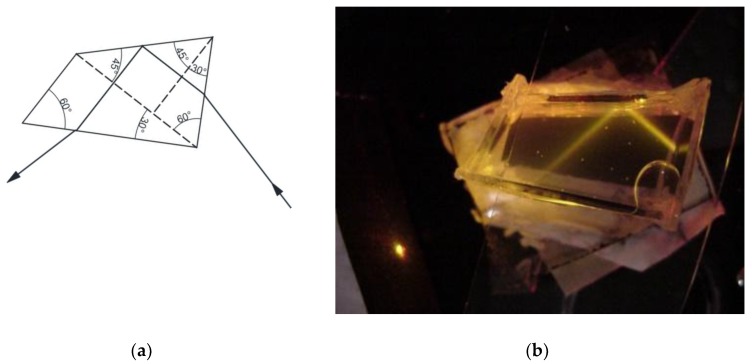
(**a**) Solid Pellin-Broca prism. Light comes from a collimator and ends in a telescope objective; and (**b**) a photograph showing one of the fabricated Pellin-Broca prims. Light trajectory inside the Pellin-Broca can be seen. Light enters from the right side, passes through the Pellin-Broca, and exits from the lower left side. On the left side a point of light can be seen; this is the outgoing light from the prism that illuminates a piece of black cardboard.

Figure 8a shows the solid Pellin-Broca optical structure along with the path that a beam of light with a certain wavelength follows [8]. The action of the prism is equivalent to that of a 60° prism at minimum deviation. If one desired wavelength is needed at the telescope reticle the prism table should be rotated. This table is calibrated to enable the wavelength to be read directly. Our proposal is to replace the solid Pellin-Broca prism by a hollow one. Instead of turning the prism to select a wavelength it is possible to change the liquid refractive index.

The hollow Pellin-Broca prism was made by cutting several pieces of plane-parallel glass plates and glued them to a base which was a flat glass. One of the fabricated prisms is shown in Figure 8b.

## 7. Testing the Pellin-Broca Prism

**Figure 9:**
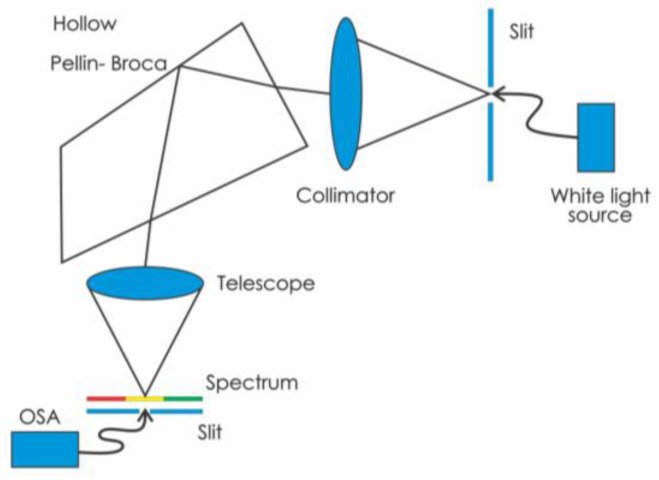
Optical configuration used to characterize the spectrum when the liquid in the hollow Pellin-Broca prisms was changed.

**Figure 10:**
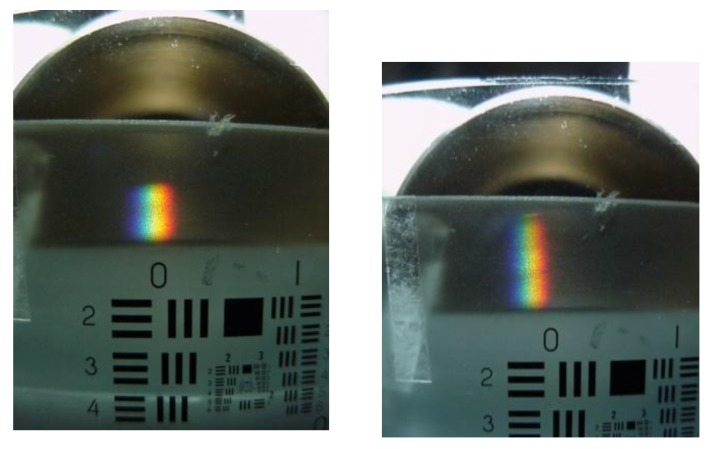
Two photographs that show the spectrum spatial position with reference to the test target when liquids with different refractive index were poured in the hollow Pellin-Broca prism. Notice that the spectrum has shifted with reference to the “0” in the chart. The refractive indices of the liquids were: 1.3325, and 1.3385.

Once the hollow prism was fabricated it was tested with the optical configuration shown in Figure 9. White light from a source was used to illuminate a slit, which was at the focal distance of a collimating lens. Then light passed through the prism and the light was collected by a second lens (telescope lens). At its focal length a spectrum was seen. If a second slit is placed at the focus of the telescope lens, a narrow section of the spectrum can be isolated. Emerging light will be almost monochromatic and the spectroscope could behave as a monochromator. To experimentally test the lateral displacement of the spectrum several liquids with the following refractive indices were used: 1.3325, 1.3335, 1.3345, 1.3355, 1.3365, 1.3375, and 1.3385. The result of the experiment can be seen in Figure 10. Each photograph shows a spectrum. Its position relative to the zero, “0”, digit shown in the USAF test target, changes as the refractive index of the liquid changes. Thus, we have probed that it is possible to select the output wavelength.

**Figure 11:**
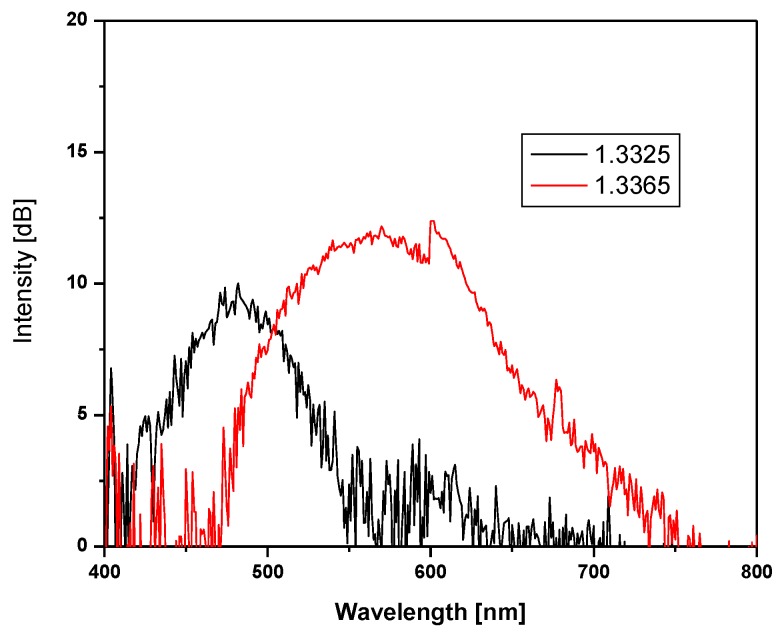
Plot showing the shift of the spectrum when different refractive indices were used in the prism.

To quantify the spectrum angular movement as a function of the liquid refractive index in the hollow prism an optical spectrum analyzer (OSA) was used. The tip of an optical fiber, with a 200 µm core diameter, was placed at the telescope slit. The other end was connected to the OSA. Two refractive indices were used, one with a refractive index of 1.3325 and the other with a refractive index of 1.3365. The plots showing the intensity as a function of the wavelength can be seen in Figure 11. A shift of the plot to the right is noticed when the refractive index increased.

## 8. Hollow Prisms Applied to the Measurement of Temperature

**Figure 12:**
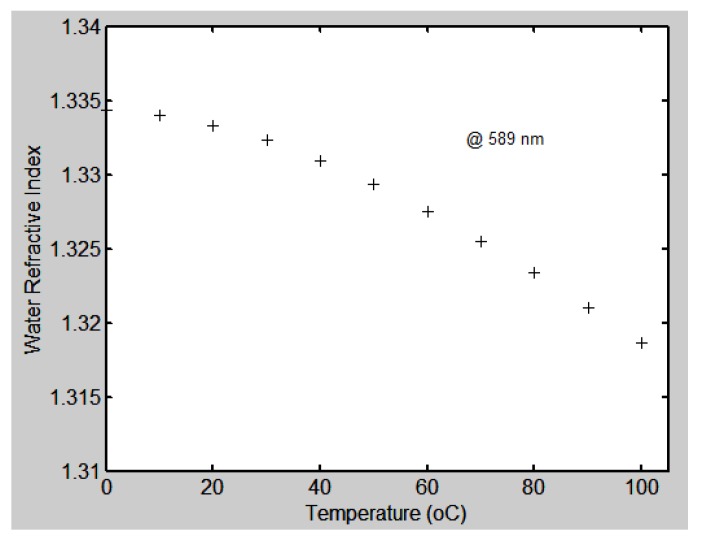
Behavior of water’s refractive index as a function of temperature (°C) [12].

Among the temperature-sensing optical methods are ones based on spectroscopy [13], coherent anti-Stokes Raman scattering (CARS) [14], fiber optics [15] and others [16,17,18,19,20,21,22]. Here we present an optical method based on the change of the refractive index of liquids. Refractive index, besides being a function of wavelength, is a function of the liquid temperature because the density of liquids decreases or increases with changes of temperature. In [12] is mentioned a study about the refractive index of water as a function of temperature and wavelength. This study is related with the use of water in the human organism. The graph in Figure 12 shows the behavior of water’s refractive index as a function of temperature when light with a wavelength of 589 nm is used [12]. We can notice that, in the range from 0 °C to 100 °C, water’s refractive index diminishes from 1.33432 to 1.31861.

**Figure 13:**
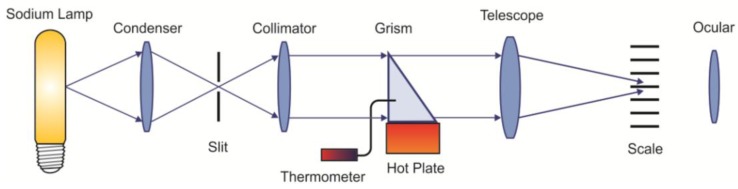
Optical configuration used to test the possibility of using the prisms as thermometers.

**Figure 14:**
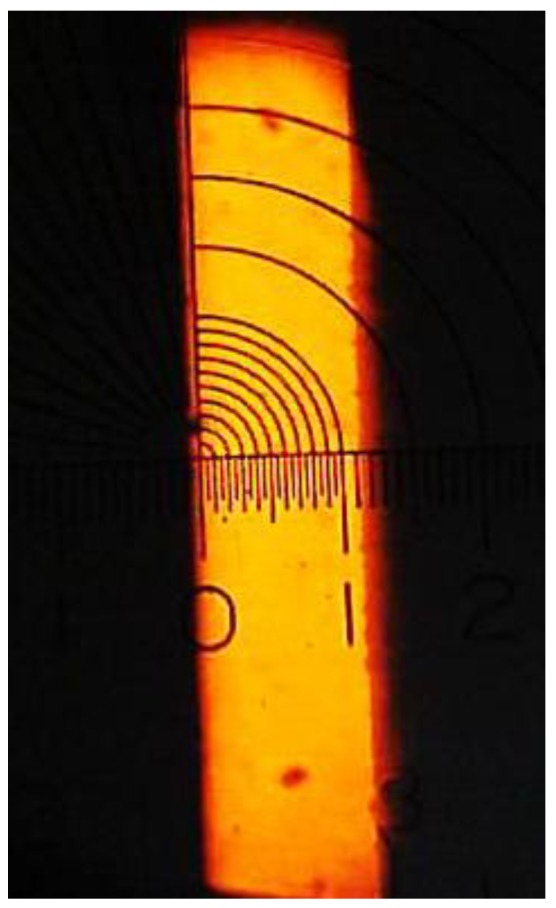
Photograph of the slit image given by the telescope lens. In the slit image plane a reticle composed of circles and a horizontal scale was placed. The minimum distance between divisions in the horizontal scale was 50 µm.

The change of the refractive index with temperature allowed us to use hollow prisms as thermometers. Liquids used in the experiments were water, glycerin, isopropyl alcohol, or mixtures of them. Figure 13 shows the implemented optical configuration. A sodium lamp was used as a light source. Through a condenser lens a slit of a collimator was illuminated. The prism was placed over a hot plate to change its temperature. For calibration purposes, a thermometer was placed in the prism (TEGAM 871A digital thermometer; accuracy: ±0.25 rdg +1 °C; resolution 0.1 °C/1°, Geneva, OH, USA). After passing through the prism the light was focused by the lens telescope and falls on a reticle where the slit image was formed (Figure 14). By means of an ocular a magnified portion of the scale was seen. Due to the increase/decrease of the liquid’s refractive index when the temperature changed, the position of the slit image on the scale moved. This position was quantified by the horizontal scale. Liquids used in the experiments are described in Table 4.

**Figure 15:**
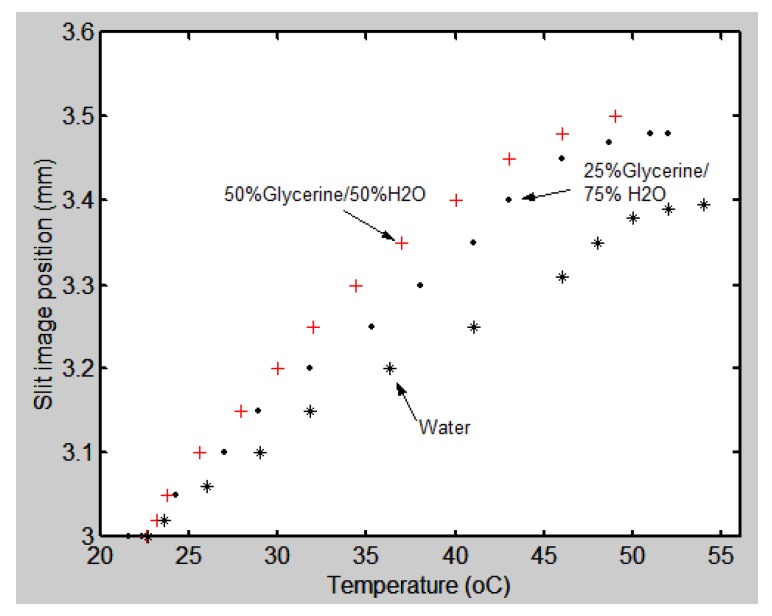
Experimental data of the slit image position as a function of temperature (°C) when a grism was used. Several liquids were used.

The first experiment was done with the grism. A calibration graph was obtained relating the slit image position (in mm) as a function of temperature (°C). The graph in Figure 15 shows the experimental results. The liquids used were water, 25% glycerin/75% H_2_O, and 50% glycerin/50% H_2_O. The density of each mixture is different. By looking at the graphs we can see that when liquids with high density are used the sensitivity of the configuration is better. The graphs reach a maximum and then stabilize. Additionally, they show a linear section. By taking two points in this section we can calculate its slope, which will be its sensitivity. For water, the sensitivity is 0.013 mm/°C, for the 25% glycerin/75% H_2_O mixture it is 0.018 mm/°C, and for 50% glycerin/50% water it is 0.021 mm/°C.

The sensitivity of the grism hollow prism in the measurement of temperature can be increased or decreased. By increasing (decreasing) the lateral displacement of the image slit, the sensitivity increases (decreases). The lateral displacement can be increased by increasing the focal length of the lens that forms the image of the slit. We have conducted some calculations with a design program considering two focal lengths, *f* = 15 cm and *f* = 30 cm, for the grism configuration that was used. Results can be seen in Table 5. We can see that the lateral displacement of the slit image increases when the focal length increases for a given refractive index (or, equivalently, a given temperature).

**Figure 16:**
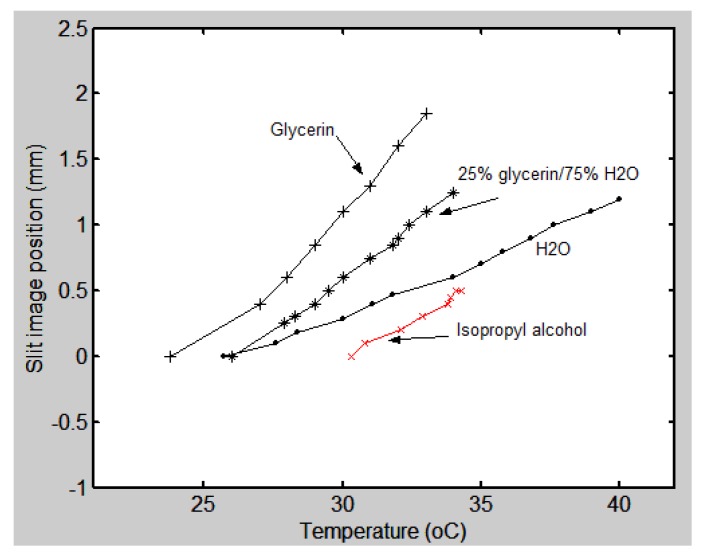
Experimental data of the slit image position as a function of temperature (°C) when a Pellin-Broca prism was used. Several liquids filled the hollow prism.

Regarding the use of a constant-deviation Pellin-Broca prism for temperature measurement, the optical configuration used was, again, the one shown in Figure 13. Instead of the grism, the Pellin-Broca was used. Thus, the trajectory was bent by 90°. Here, besides water and glycerin, or mixtures of them, isopropyl alcohol was also used. The results can be seen in Figure 16. In the case of isopropyl alcohol, the graph shows a tortuous behavior. We think this is due to the fast evaporation of the alcohol. This evaporation would cause the temperature to change unevenly. The hollow prism had a glass cover but a small space of about 2 mm × 6 mm was left to insert the thermocouple used in the calibration process. Through that space alcohol evaporated. Again, by looking the graphs we notice that when more glycerin is present in the mixture the sensitivity increases. By considering the linear section in each curve and taking two points from them we can calculate the slope, which is the sensitivity. For the water we have 0.086 mm/°C; for 25% glycerin/75% H_2_O, 0.16 mm/°C; and for glycerin, 0.4 mm/°C. The graph for glycerin in this linear portion also shows us that a minimum change in temperature of 1 °C can be detected.

The image slit position could be difficult to read when large temperatures, above 50 °C, and liquids with high densities are present. Results are not consistent. There are convection currents in the liquids that degrade the slit image due to the passage of light through these currents. To avoid these convection currents the use of a method that circulates the liquid in the cell is suggested.

**Figure 17:**
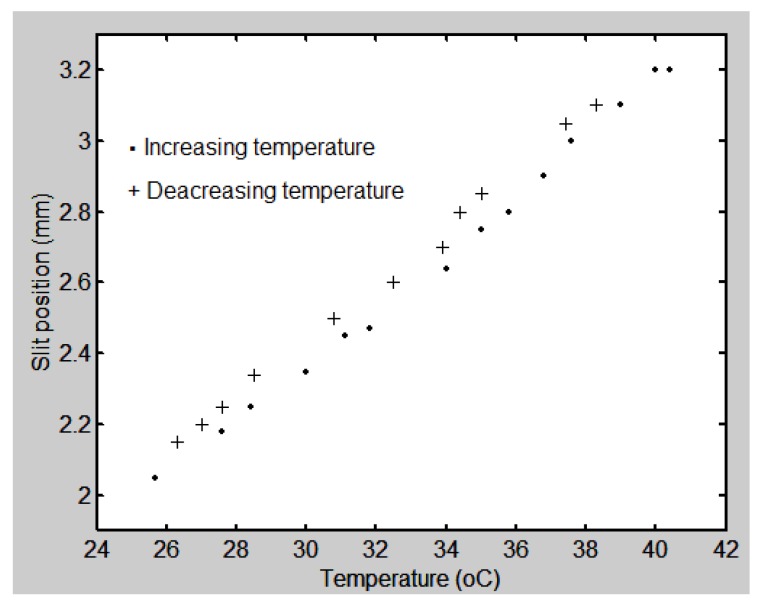
Experimental data showing the behavior of the slit position as a function of temperature when it increased and decreased. A Pellin-Broca prism was used.

Regarding the hysteresis behavior of the prisms, here we describe an example with the Pellin-Broca prism. In the experiment, the starting temperature was about room temperature. Then it increased until it reached about 40 °C. At that stage the hot plate was left to cool. The slit position behavior can be seen in Figure 17.

## 9. Conclusions and Comments

We have shown that it is possible to fabricate hollow grisms that cover a spectral band in the visible region. Central or undeviated wavelengths can be selected by changing the liquid in the grism. Thus it is not necessary to fabricate several grisms to select the central wavelength. Regarding the spectral range it can be selected by using liquids with different refractive indices.

Additionally, we have shown that a hollow Pellin-Broca prism can be used to disperse light. When used in a constant deviation spectroscope it is possible to select the central output wavelength by changing the liquid in the prism. That is, it is not necessary to rotate the prism, as is commonly done.

The use of hollow prisms as thermometers was also shown. A calibration graph was obtained relating the position of the slit image as the liquid temperature changed. Slit image position was found visually with the help of an ocular. To perform this reading automatically we suggest using a CCD or CMOS linear or matrix sensor. Some CCDs have pixels of about 5 µm × 5 µm with a distance between pixels of a few microns. Thus, the edge of the slit could fall on a CCD sensor and its position could be quantified.

The temperature measurement methods with the two hollow prisms present different characteristics. The main results are shown in the graph of Figure 15 for the grism and graphed in Figure 16 for the Pellin-Broca. The best sensitivity for the grism was 0.021 mm/°C and for the Pellin-Broca 0.4 mm/°C. Thus, if one needs good sensitivity in a range between 27 °C to 32 °C the second prism filled with glycerin should be selected. However, if the required sensitivity is low, but the range is between 26 °C and 40 °C, it is useful to use the Pellin-Broca filled with water. Optical configurations of both hollow prisms are versatile because it is possible to change parameters like the density and refractive index of the liquid, and/or the focal length of the lens in the telescope. With these parameters we can adapt the optical setup to our needs.

Regarding the time to reach stability when a measurement is conducted depends on the size of the prism and the liquid used. Considering that the prisms have dimensions of several centimeters and that the liquids used were water, isopropyl alcohol, and glycerin, the time for a thermal stability condition was about 1.5 min when temperatures changed by a few degrees. However, in the setups presented, the response time was about 30 s. These lapses of time could be shortened if the prisms are smaller or the liquids and the materials with which the prisms are made have a smaller thermal inertia.

## Figures and Tables

**Table 1:** Results given by an optical design program.

Refractive Index *n*	Deviation Angle for 1st Diffractive Order (Degrees). *λ* = 400 nm
1.33	0.081
1.34	0.653
1.35	1.258
1.36	1.869
1.37	2.487

**Table 2:** Here are shown the wavelengths that passed undeviated when the liquid in the hollow grism was changed. The second column shows the experimental value taken from Figure 7. Values in the third column were calculated with Equation (1) and values in last column were obtained by means of an optical design program that simulated the experimental grism.

*n*	Experimental Peak Wavelength (nm) Taken from Figure 7	Theoretical Undeviated Wavelength (nm). Equation (1)	Optical Design Calculated Wavelength (nm) that Passed Undeviated
1.33	402.5	401.2	401.2
1.34	410	413.3	413.3
1.35	422	425.5	425.5
1.36	432	437.6	437.6
1.37	446	449.8	449.8

**Table 3:** Transmitted undeviated light as a function of the change in the refractive index due to a temperature change.

Temp (°C)	*n* Ref. Index	*λ* Undeviated
10	1.33408	406.1
20	1.33336	405.3
30	1.3323	404.0
40	1.3309	402.3
50	1.3293	400.3
60	1.3275	398.1
70	1.3255	395.7
80	1.3234	393.1

**Table 4:** Liquids used in the experiments and some characteristics of them [23].

Liquid	Density g/mL @ 25 °C	Refractive Index @ 589 nm
Water	1	1.33336
Glycerin	1.259	1.472
Isopropyl Alcohol	0.785	1.375

**Table 5:** Lateral displacement of the slit image when two focal distances were considered.

Water Refractive Index @ 401.2 nm *n*	Displacement (mm) *F* = 15 cm	Displacement (mm) *F* = 30 cm
1.3343	−0.67	−1.34
1.33336	−0.52	−1.04
1.3323	−0.36	−0.72
1.33	0	0
1.3275	0.39	0.78
1.3210	1.39	2.78
